# Hamular bursitis as a cause of orofacial pain. A case report

**DOI:** 10.4317/jced.60702

**Published:** 2023-07-01

**Authors:** Blanca Prats-Sisquella, Nikol A. Peeva, Adrià Jorba-García, Tomas Escuin, Rui Figueiredo, Jose-Javier Bara-Casaus

**Affiliations:** 1 DDS. Fellow of the Master’s degree programme in Oral prostheses and rehabilitation, Faculty of Medicine and Health Sciences, University of Barcelona. Spain; 2 DDS, MS. Master’s degree in Oral surgery and implantology, Faculty of Medicine and Health Sciences, University of Barcelona, Spain; 3 MD. Department of Oral Rehabilitation and Maxillofacial Prosthesis, School of Medicine and Health Sciences, Universitat de Barcelona, Barcelona, Spain; 4 DDS, MS, PhD. Oral Surgery and Implantology, Faculty of Medicine and Health Sciences, University of Barcelona, Barcelona, Spain; 5 EBOMS, MD, DDS, PhD: Director of the Maxillofacial Institute of the University Hospital Sagrado Corazon (Barcelona), España. Co-director of the postgraduate programme temporomandibular join and orofacial plain, Faculty of Medicine and Health Sciences, University of Barcelona, Spain

## Abstract

Pterygoid hamular bursitis is an infrequent cause of orofacial pain due to a hypertrophic pterygoid hamulus. The pain is often referred to the pharynx, temporomandibular region and facial zone, and requires a differential diagnosis with other craniofacial entities. This paper describes a patient with pterygoid hamular bursitis that presented pain of neuropathic characteristics in the left retromolar region, associated with odynophagia and temporomandibular joint disorders. Based on the clinical and radiological findings, a surgical resection of the pterygoid hamulus was decided. After the surgical procedure the patient still reported symptoms so additional specific treatments such as peripheral nerve block and infiltration were performed. Four months later, the patient developed a squamous cell carcinoma on the left margin of the tongue, which was surgically treated. At present (thirty months follow-up), the patient has no pain or signs of tumor relapse.

** Key words:**Pterygoid hamular bursitis, orofacial pain, oral carcinoma, temporomandibular joint disorders, radiofrequency, neuropathic pain.

## Introduction

Pterygoid hamular bursitis is an infrequent cause of orofacial pain referred to the palatine and pharyngeal regions, and is attribuTable to a hypertrophic pterygoid hamulus. The hamulus or pterygoid hamular process is an elongated, hook-shaped bone eminence located in the medial pterygoid plates of the sphenoid bone. It is located posterior and medial to the maxillary tuberosity, is projected in a caudad and anterolateral direction, and constitutes an insertion point for a number of muscles and ligaments (1).

The tendon of the muscle elements that regulate tension of the soft palate and dilatation of the Eustachian tube runs along the hamular process; these muscles contract when the patient speaks, swallows, chews, inhales, sneezes and/or yawns (1). The tendon is surrounded by a fibrous bursa that reduces friction upon the hamular process during action of the abovementioned muscles. As a result, inflammation of the bursa would cause local or referred pain each time tension increases in the soft palate (2,3).

The etiopathogenesis of hamular bursitis is unclear, although it has been reported to occur following trauma. In other cases, bursitis may be due to hypertrophy of the pterygoid process, which makes the region more vulnerable to trauma (4,5).

In many cases, a palpable and firm prominence is noted in the region of the soft palate. Clinically, pterygoid hamular bursitis manifests as a burning sensation, inflammation and sometimes erythema of the mucosa in the region of the pterygoid process. The pain often irradiates to the pharynx, the temporomandibular region and to other facial zones (4). Thus, an accurate differential diagnosis with other conditions like otitis media, temporomandibular joint dysfunction (6), glossopharyngeal neuralgia, headache, dental infections (pericoronaritis) (1,2), Ernest syndrome and Eagle syndrome (7) is paramount.

The present paper describes a case of pterygoid hamular bursitis associated to facial pain in a patient who posteriorly developed a homolateral squamous cell carcinoma of the tongue.

## Case Report

A 69-year-old woman (profession: anesthesiologist) reported to the maxillofacial clinic due to a non-painful altered sensation in the lower left lingual molar region that started four months before.

The patient’s medical history included hypothyroidism, mild mitral valve prolapse and cardiac arrhythmia. The patient was receiving levothyroxine 25 µg, as regular medication.

The physical examination revealed pain in response to palpation of the temporomandibular joint and left-side masticatory muscles, with referral to the homolateral submaxillary zone. The initial intraoral examination evidenced plaque accumulation, while the panoramic X-ray study showed no relevant alterations. A provisional diagnosis of arthralgia and myofascial pain was established, following the Diagnostic Criteria for Temporomandibular Disorders (DC/TMD) Classification of 2014 (8).

A local anesthetic infiltration (1.8 ml mepivacaine 3%) on the trigger points of the left masseter and mylohyoid muscles was performed. The occlusal splint provided by the patient was adjusted, and diazepam (5 mg) was prescribed, with referral to the osteopath for supporting treatment.

After two months, the pain increased in intensity (9/10 on the visual analog scale (VAS)) and acquired neuropathic features, with the patient referring a burning sensation in the lower left retromolar region, extending to the homolateral mandibular zone. Eating acted as a trigger point intensifying the pain and initiating odynophagia that severely impaired the swallowing function.

**Figure 1:**
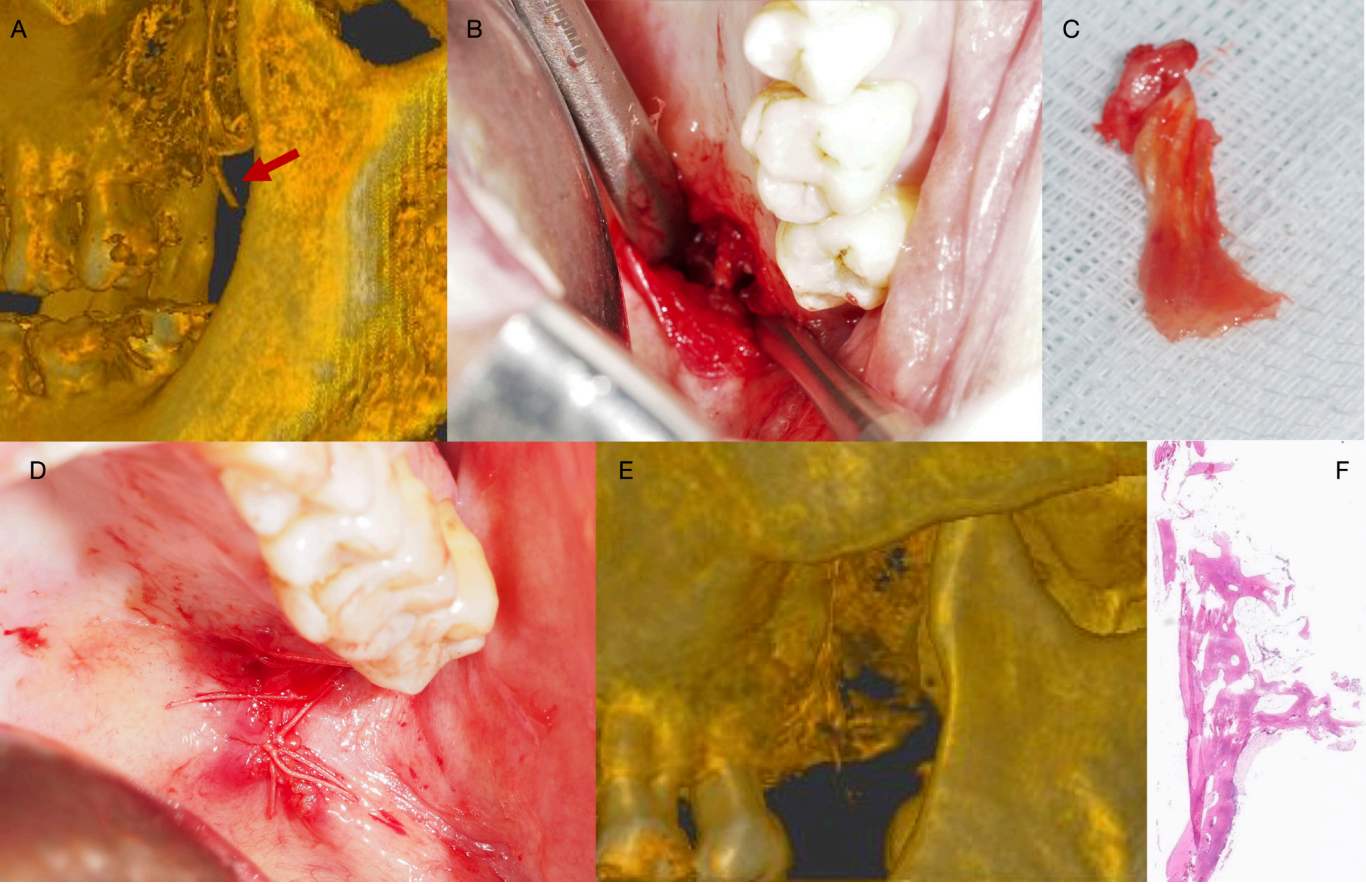
Surgical aspects of the case. A. Three-dimensional CBCT view of the maxillo-mandibular region. Note the hypertrophic pterygoid process. B. Direct transoral approach. C. Fragment of the left pterygoid process, with tissue of irregular morphology, measuring 15 x 3 x 2 mm. D. Incision closure with reabsorbable suture. E. Three-dimensional CBCT view of the maxillo-mandibular region following removal of the pterygoid process. F. Microscopic characteristics following resection of the pterygoid process. Fragment of trabecular bone with adipose marrow tissue (hematoxylin - eosin; original magnification x 2).

During the intraoral examination, the internal left pterygoid process was found to be more prominent than its opposite counterpart, and the cone-beam computed tomography (CBCT) confirmed a hypertrophy of the pteroygoid process (Fig. 1A).

A diagnostic infiltration with triamcinolone acetonide 40 mg/ml (8 mg) and 2% mepivacaine (0.5 ml) was performed, that allowed to eliminate the pain. Based on the clinical and radiological findings, a provisional diagnosis of pterygoid hamular bursitis was established.

Thus, sectioning and removal of the left pterygoid process hook was carried out under general anesthesia. The operation and postoperative course were uneventful (Fig. 1), and the histological study confirmed a bone fragment with normal characteristics.

After two weeks the pain when swallowing had disappeared, but the mandibular discomfort (VAS score 5/10) and trigger point pain persisted (VAS score 7/10). The patient was advised to use the splint for as many hours as possible, and paracetamol with methocarbamol (Robaxisal®)(2 Tablets/8 h) and pregabalin (Lyrica®)(75 mg/12 h) were prescribed. Forty-eight hours later, botulin toxin (Botox®)(20 IU) was infiltrated in the internal pterygoid muscle. After two weeks, the clinical condition had improved.

Forty-five days after the above-mentioned treatment, the pain had a VAS score 4/10, and oxcarbazepine (Tryleptal®)(60 mg/ml) was prescribed since pregabalin was not well tolerated. Left third trigeminal branch block was performed with 0.25% bupivacaine (3 ml) plus 20 mg of triamcinolone – repeating the procedure three times in the course of a month. Posteriorly, pulsed radiofrequency therapy was applied (two cycles of 120 seconds each) in peripheral branches of the mandibular nerve (V3). Lastly, 225 mg of lidocaine were administered as an intravenous perfusion. The final pain VAS score was 1/10.

**Figure 2:**
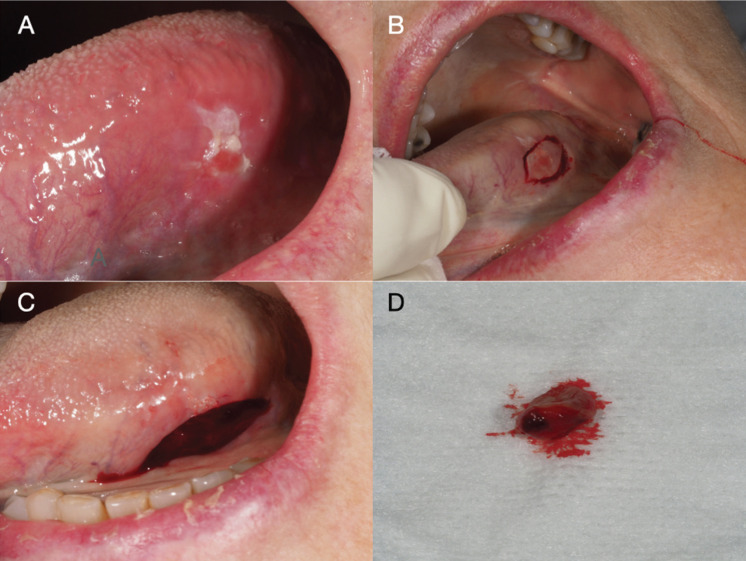
Aspects of the tongue lesion. A. An erythroleukoplastic lesion located on the left lateral margin of the tongue. B. Incision using a scalpel (#12 blade). C. The lesion following excisional biopsy. D. The biopsy fragment measuring 9 mm in size.

**Figure 3:**
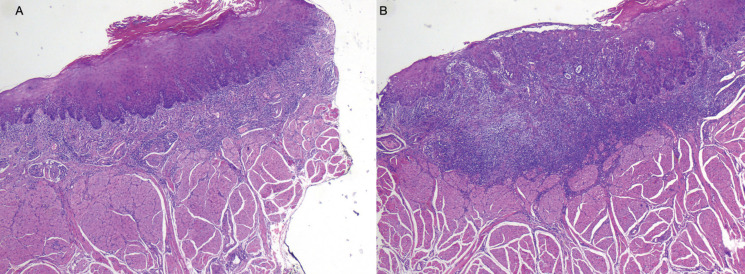
Microscopic characteristics of the tongue lesion. A, B. Mucosal tissue sections showing a neoplastic proliferation of epithelial cells forming nests and cords that infiltrate the dermis, with squamous cell differentiation and moderate anaplasia. Mucosal dysplasia is observed at the lateral margins of the lesion (hematoxylin - eosin; original magnification x 2).

Two weeks later, the patient attended the maxillofacial clinic, and an indurated lesion, with irregular margins and with an erythematous and leukoplakia-like appearance was identified on the left lateral margin of the tongue (Fig. 2). The incisional biopsy revealed a moderately differentiated squamous cell carcinoma. The lesion was surgically removed, and the histopathological study confirmed the diagnosis, with tumor-free resection margins and no vascular or perineural invasion (Figs. 2,3). No alteration in adenopathies were found in the ultrasound and radiographic studies.

The reported pain disappeared entirely after three months (VAS score 0/10). Thirty months the surgical treatment, the patient was asymptomatic, with no signs of relapse following removal of the tongue carcinoma.

## Discussion

Pterygoid hamular bursitis cases may be challenging to diagnose. Different craniofacial pain conditions can exhibit similar symptoms – a fact that may delay the diagnosis and the treatment. In the present case report, an initial diagnosis of a temporomandibular joint disorder was initially established.

Our patient presented symptoms associated to glossopharyngeal nerve involvement, such as odynophagia, which increased when swallowing. This may be explained by the presence of a long or wide pterygoid hamulus, that might cause a mechanical injury to the surrounding tissues, which in turn can lead to inflammation or fibrosis of the bursa. Such situations can affect branches of the trigeminal, glossopharyngeal and facial nerves – producing pain in the palate, pharynx, face and neck region (9).

Anatomically, the pterygoid process of the sphenoid bone is located within the infratemporal fossa. Considering the main structural elements within the infratemporal fossa, mention must be made of part of the innervation of three cranial nerves: the mandibular (V3), the glossopharyngeal (IX) and the facial nerves (VII). The chorda tympani nerve (VII) joins with the posterior margin of the lingual nerve (V3). Likewise, the lesser petrosal nerve (originating from the IX nerve), which forms part of the tympanic plexus of the middle ear, joins with the mandibular nerve (V3). The complex clinical manifestations of the pterygoid hamular bursitis could be explained by the innervation of all the implicated anatomical structures, giving rise to symptoms associated with temporomandibular joint disorders and otorhinolaryngology pathologies (2,6).

With regard to hamular bursitis and temporomandibular joint dysfunction, the pain produced by the bursa has been related to the muscle activity, since the muscle elements that tense the soft palate and eardrum are activated when chewing and swallowing. In the presence of pain, greater protective co-contraction activity may be generated, causing temporomandibular joint dysfunction5. The pain sensation is typically reproduced on applying continuous pressure to the hamular region. Improvement of the pain following diagnostic infiltration of the hamulus with local anesthetic could be suggestive of bursitis (6). Cone-beam computed tomography is the imaging technique of choice for establishing a clearer diagnosis, since it can reveal possible fractures of the hamular process, osteophytes (10) or signs of hypertrophy of the pterygoid process (7) - which according to Putz and Kroyer (1999), measures 7.2 mm in length on average (11). In our patient, the surgically removed pterygoid process fragment measured 15 mm in length (Fig. 1C).

In our patient, the progression of the symptoms from nonspecific discomfort in the lower left retromolar region to odynophagia and homolateral facial pain of neuropathic characteristics (VAS score 9/10) caused us to modify our initial diagnostic impression and suspect the presence of some other disease process concomitant to temporomandibular joint dysfunction. Accordingly, following clinical exploration, with clear palpation of the hook of the pterygoid process, a CBCT was requested, revealing hypertrophy of the left pterygoid hamulus. Based on this observation and temporary pain cessation following corticoid and local anesthetic infiltration, we suspected the existence of hamular bursitis. Since the symptoms failed to resolve with medical treatment, surgical removal of the hamulus was decided (1,7).

There is no agreement regarding treatment, though conservative management is generally advised as a first step, combining the elimination of irritative factors with medical treatment in the form of corticosteroid injections in the region (9), with the possibility of adding nonsteroidal antiinflammatory drugs. If the symptoms fail to subside, surgical resection of the hamulus with the removal of osteophytes or of the bursa is indicated. If inflammation or fibrotic elements are found, the bursa is eliminated, leaving the tendon of the soft palate muscle intact (4,6).

Following surgery, our patient experienced complete resolution of her pain when swalling, though a persistent pain of neuropathic characteristics was present in the zone innervated by V3. In order to avoid possible central sensitization and thus chronification of neuropathic pain, the decision was made to administer specific coadjuvant treatments in the Pain Unit, such as block and radiofrequency therapy of the peripheral branches of V3, together with intravenous lidocaine. There is scientific evidence of the effectiveness of the intravenous administration of this kind of local anesthetic in the management of chronic pain (12). The use of pulsed radiofrequency therapy was justified in view of its favorable outcomes, with fewer complications versus conventional radiofrequency therapy - such as sensory paresthesias or masseter muscle paralysis - due to the emission of large heat doses associated with the latter technique (13). Future research is required to establish the mechanisms of action, appropriate dose, duration and forms of the abovementioned treatments.

The patient posteriorly developed a squamous cell carcinoma of the left lateral margin of the tongue. A possible explanation for this is that the hemimandibular dysesthesia and dystonia secondary to the initial treatment may have increased trauma to this area. Parafunctional habits such as nibbling or suction of the oral mucosa, tongue interposition or atypical swallowing, may be responsible of mechanical irritation. In this regard, chronic mechanical irritation has been regarded as a potential risk factor for the development of oral malignancy (14). Another factor that could be related to the sudden appearance of squamous cell carcinoma could be immune suppression due to the stress caused by such a painful clinical condition (15).

Due to its clinical diversity, hamular bursitis is difficult to diagnose. The superficial location of the bursa predisposes it to chronic inflammation and/or trauma. In turn, the proximity of different nerve structures can produce orofacial pain in different regions, giving rise to diagnostic error and delays. A multidisciplinary approach could facilitate earlier patient recovery and thus prevent chronification of the pain, which would imply greater complexity of treatment. For this reason, hamular bursitis should be included within the differential diagnosis of orofacial pain conditions.
